# Switchable Wettability of Poly(NIPAAm-*co*-HEMA-*co*-NMA) Coated PET Fabric for Moisture Management

**DOI:** 10.3390/polym12010100

**Published:** 2020-01-04

**Authors:** Shamik Chaudhuri, Chang-Mou Wu

**Affiliations:** 1 Department of Materials Science and Engineering, National Taiwan University of Science and Technology, Taipei 10607, Taiwan; shamikjumet@gmail.com; 2 Research and Development Center for Smart Textile Technology, National Taipei University of Technology, Taipei 10608, Taiwan

**Keywords:** smart polymers, thermoresponsive, lower critical solution temperature, wettability, smart textile

## Abstract

In this study, we synthesized a random poly[(*N*-isopropylacrylamide)-*co*-(2-hydroxyethylmethacrylate)-*co*-(*N*-methylolacrylamide)] [poly(NIPAAm-*co*-HEMA-*co*-NMA)] copolymer through free-radical polymerization. The NIPAAm, HEMA and NMA moieties were framed to provide thermoresponsiveness, water absorption and retention control, and chemical cross-linking to achieve stability in aqueous medium, respectively. The copolymer showed a significant change in optical transmittance with a variation in temperature due to the change in volume (i.e., hydrophilic/hydrophobic) between 25 °C and 40 °C, attributed to the lower critical solution temperature property of the NIPAAm moiety. The copolymers were wire-bar-coated onto polyethylene terephthalate (PET) fabric. Variation in the water contact angle affirmed the switchable wettability due to the change in temperature. We tested the coated fabrics for moisture absorption and release at different temperatures. The results at 20 °C and 37 °C indicated that the P2 copolymer had the highest moisture absorption and release capability. Therefore, the copolymers with tailored properties can be used as smart textiles for activity specific clothing.

## 1. Introduction

The rapid development of materials in science and engineering generates novel materials with unique properties for complex applications. Among them, the most emerging materials of this century are smart and intelligent materials that are highly responsive to a minute change in the surrounding environment [1]. We can find many examples of smart materials around us, like the leaves of *Mimosa pudica* (highly sensitive when touched) or the leaflets of *Codariocalyx motorius* (sensitive to sunlight) [2]. Inspired by those, scientists and researchers have endeavored to synthesize materials that can sense, react, and adapt to their environment, having scientific and industrial significance. Polymers play an important role in smart materials. There are various types of external stimuli that affect the properties of stimuli-responsive polymers, such as ion concentration, pH, and temperature [3,4].

Temperature responsive polymers are widely applied in wearable electronics, drug delivery, temperature-based separation, smart textiles, etc. [5,6,7,8]. Some natural thermoresponsive polymers are cellulose, starch, xanthan gum, and carrageenans whereas artificial thermoresponsive polymers are poly *N*-isopropylacrylamide (PNIPAAm), polyoxazoline, poly(2-(dimethylamino)ethylmethacrylate), and pluronic F127. Among such polymers, PNIPAAm displays a characteristic lower critical solution temperature (LCST) at 32 °C, close to the human body temperature [9]. This LCST is basically a transition temperature. Thermoresponsive polymers below LCST are soluble in water but become partially insoluble when temperature is increased above it. From thermodynamic aspect, we can consider that Gibb’s free energy (∆G = ∆H − T∆S) for mixing is negative when polymer dissolves in water at below LCST. Such phenomenon can be held when the enthalpy of mixing (∆H) will be negative. This can be physically interpreted as H-bonding between polymer and water molecules. Another condition will be the negative entropy (∆S) for such dissolution. However, the free energy value becomes positive when temperature increases above LCST. At higher temperature, enthalpic H-bonding will be reduced and entropy term (T∆S) becomes predominant leading to a positive Gibb’s free energy of mixing. This is attributed to the phase separation phenomena and manifested LCST-phase transition as entropy-driven. This characteristic makes PNIPAAm the most intensively explored thermoresponsive material for biomedical and textile applications [10]. PNIPAAm shows reversible coil-to-globule transition (CGT) phenomena in aqueous solutions when temperature varies below and above the LCST. Below the LCST, the hydrophilic amide groups form hydrogen bonds (H-bonds) with water molecules resulting in a swollen structure, whereas above the LCST, the hydrophobic isopropyl groups interact stronger and the H-bonds are broken resulting in a compact, shrunk structure [2,11]. By incorporating hydrophilic or hydrophobic monomers, the LCST can be tailored for particular applications [12]. Therefore, the concept of LCST is highly desirable to design any new thermoresponsive copolymer. Additionally, hydrophilicity or hydrophobicity of a copolymer can be tailored by tuning its LCST value. LCST is most important for thermoresponsive polymers for understanding mixing phenomena especially in aqueous solution. LCST can be tuned based on the desired architecture of the polymer, such as hydrogels, particles, micelles, interpenetrating network, etc. The theoretical concept of LCST was first established as Flory–Huggins solution theory. This is a lattice-fluid (LF) model which considered the dissimilarity in molecular sizes. The volume fraction and number of moles was considered with a parameter denoting energy of interspersing polymer and solvent molecules at constant temperature and pressure, and this value was expressed as a change of Gibb’s energy. However, there are some limitations. A more advanced theory was introduced later as Flory–Krigbaum theory. Further, scaled particle theory (SPT) is considered to model LCST-based CGT for isolated polymer chains in highly diluted solution. Some other theoretical approaches are quite important such as Debye–Huckle approximation for long range ion–ion interaction and Veytsman statistics for short range ion–dipole interaction in aqueous polymer solution with free salts.

Some research groups adopted this LCST property in smart textile applications. Hu et al. modified a smart textile for wound dressings by graft copolymerization of NIPAAm and polyurethane onto cellulose/PET. They achieved the tunability of water absorption by varying the temperature and pH [13]. Bashari et al. investigated the water retention capacity of a PNIPAAm/chitosan modified cotton fabric [14]. Chen et al. copolymerized PNIPAAm with N-hydroxymethyl acrylamide (NHMAAm) and grafted it on cotton fabric. The thermosensitivity and wettability of the material were investigated [15]. Yang et al. also analyzed the structure and molecular mobility of a PNIPAAm grafted cotton fabric using ^1^H NMR spectroscopy [16]. Despite such progress, many challenges remain.

The thermoresponsiveness of NIPAAm can be utilized as a driving force for moisture absorption and release in thermoregulatory textiles for body comfort. Garments create a microclimate, situated between human skin and the first layer of cloth. The human body and clothing support a microclimate following certain mechanisms to maintain the proper body temperature. First, the body produces metabolic heat in the internal layer of the skin to circulate sweat. Second, sweat is produced to cool the body, and finally, this sweat, in the form of liquid or vapor (sensible and insensible perspiration), has to get through the garment [17]. The first two mechanisms are uncontrollable. The third mechanism can be controlled by balancing the microclimate with the outer climate using thermoregulatory smart textile clothing with moisture management capabilities. Perspiration generates a sticky sensation that discomforts the wearer, so the textile needs to transmit heat and moisture from the body to the outer environment rapidly. This rapid transmittance would halt the increase in the body temperature and result in a more comfortable state. Thermoregulation with phase change materials or shape memory polymers has been reported; however, we have yet to explore NIPAAm-based stimuli-responsive polymers for this particular application [18,19,20]. Thus, the objective of our work is exploring smart thermoregulatory materials with high capacity for moisture absorption and release.

Poly(2-hydroxyethylmethacrylate), PHEMA is a widely used material which shows excellent water absorption and retention controlling property at different conditions. Additionally, it is biocompatible and has no harmful effects on the human body. So, it can be a potential material for moisture management application in textiles. On the other hand, poly(*N*-methylolacrylamide), PNMA is a well-known cross-linker material. PNIPAAm is highly soluble in aqueous media. So, for the practical application related to moisture management, it is worthwhile to prepare a material which will not dissolve. PNMA can cross-link with other molecules by the terminal hydroxyl group. Some researchers already reported efficient utilization in design and fabrication of copolymers for the application in aqueous media and achieved good results for drop-cast film and electrospun nanofibers [21,22].

In this study, we synthesized a random poly(NIPAAm-*co*-HEMA-*co*-NMA) copolymer by free-radical copolymerization. The NIPAAm, HEMA, and NMA copolymer moieties were responsible for temperature control, water absorption and retention, and chemical cross-linking, respectively. Different molar ratios of the moieties successfully tailored the LCST. The copolymer was characterized using proton nuclear magnetic resonance (^1^H NMR) spectroscopy, Fourier-transform infrared (FTIR) spectroscopy, thermogravimetric analysis (TGA), and UV-Vis absorption spectroscopy. PET fabrics coated with copolymers and cross-linked were evaluated by contact angle analysis, moisture absorption and release tests below and above the LCST and mechanical property testing.

## 2. Materials and Methods

### 2.1. Materials

*N*-isopropylacrylamide, NIPAAm (99%), and 2-Hydroxyethyl methacrylate, HEMA (97%), were provided by Acros, Morris Plains, NJ, USA. HEMA was stored at 4 °C before usage and passed through an aluminum oxide (Al_2_O_3_) column for purification. N-methylolacrylamide, NMA (>98%) and 2,2′-Azobis(2-methylpropionitrile), AIBN (>99%) were purchased from Tokyo Chemical Industry Co., Tokyo, Japan, and Aencore, Surrey Hills, Australia, respectively. AIBN was recrystallized with an ethanol solution prior to usage. *N*,*N*-dimethylformamide, DMF (99.8%) was purchased from Echo, Miaoli, Taiwan.

### 2.2. Synthesis of Poly(NIPAAm-co-HEMA-co-NMA)

**Scheme 1:**
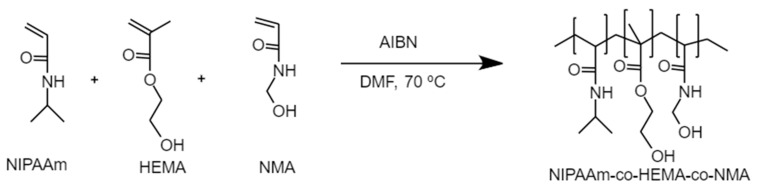
Synthesis reaction of Poly(NIPAAm-*co*-HEMA-*co*-NMA).

The random poly(NIPAAm-*co*-HEMA-*co*-NMA) copolymer was synthesized by free radical polymerization of NIPAAm, HEMA, and NMA monomers as illustrated in Scheme 1. A similar synthesis process has been reported [23,24]. The three different monomer ratios of this tri-copolymer are denoted as P1, P2, P3, and enlisted in Table 1. We considered 50, 70, and 90% NIPAAm for the samples. HEMA: NMA ratio was fixed at 3:1 for all the samples to achieve successful cross-linking. Chen et al. and Jeng et al. worked with HEMA and NMA considering different molar ratios (3.35:1 and 3.3:1) and achieved sufficient cross-linking [23,25]. The NIPAAm, HEMA, NMA monomers, and AIBN (initiator) were dissolved in DMF in a two-necked round-bottomed flask. Nitrogen gas was bubbled through the mixture for 30 min for degassing, and the mixture was placed in an oil bath at 70 °C for 24 h. The polymerization reaction was terminated by air exposure. Subsequently, the resultant solution was poured and precipitated into ether, and filtered and dried at 35 °C under vacuum.

#### 2.2.1. Synthesis of P1

A reaction mixture of 2263.2 mg (19.97 mmol) of NIPAAm, 1952 mg (14.99 mmol) of HEMA, 505.5 mg (4.99 mmol) of NMA, 16.4 mg (0.01 mmol) of AIBN, and 20 mL of DMF was used to obtain a solid white polymer (61% yield). ^1^H NMR (*d*-DMSO, 600 MHz): δ (ppm): 0.7–2.1 (peak a, b, c, f), 3.5–3.6 (peak h), 3.7–4.1 (peak e, g), 4.4–4.7 (peak k), 4.8–5.1 (peak i), 5.3–5.6 (peak l), 7.1–7.6 (peak d), 7.9 (peak j).

#### 2.2.2. Synthesis of P2

A reaction mixture of 3168.5 mg (28.01 mmol) of NIPAAm, 1171.2 mg (8.99 mmol) of HEMA, 303.3 mg (2.99 mmol) of NMA, 16.4 mg (0.01 mmol) of AIBN, and 20 mL of DMF was used to obtain a solid white polymer (64% yield). ^1^H NMR (*d*-DMSO, 600 MHz): δ (ppm): 0.8–2.1 (peak a, b, c, f), 3.5–3.6 (peak h), 3.7–4.1 (peak e, g), 4.4–4.6 (peak k), 4.9–5.2 (peak i), 5.3–5.5 (peak l), 7.1–7.6 (peak d), 7.9 (peak j).

#### 2.2.3. Synthesis of P3

A reaction mixture of 4073.7 mg (35.97 mmol) of NIPAAm, 390.42 mg (2.99 mmol) of HEMA, 101.1 mg (0.99 mmol) of NMA, 16.4 mg (0.01 mmol) of AIBN, and 20 mL of DMF was used to obtain a solid white polymer (51% yield). ^1^H NMR (*d*-DMSO, 600 MHz): δ (ppm): 0.8–2.1 (peak a, b, c, f), 3.5–3.6 (peak h), 3.7–4.0 (peak e, g), 4.4–4.6 (peak k), 4.9–5.2 (peak i), 5.3–5.5 (peak l), 7.0–7.6 (peak d), 7.9 (peak j).

### 2.3. Fabrication of Copolymer Coated PET Fabrics

**Figure 1:**
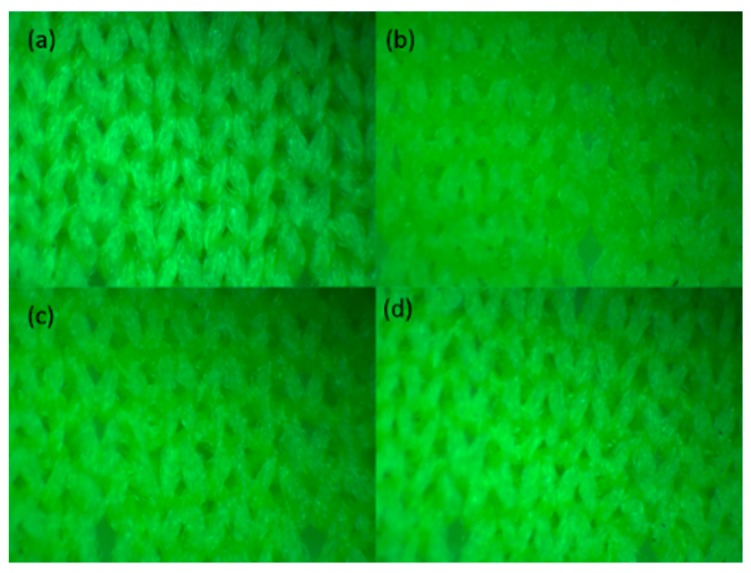
Optical microscopic images of (**a**) uncoated; (**b**) P1; (**c**) P2 and (**d**) P3 coated fabric.

The copolymer solution of 30 wt % concentration was prepared in DMF by stirring for 3 h and coated onto PET fabric by an Aaron drawdown wire bar. The dimensions of each sample were 5 cm × 5 cm. The as-prepared PET fabrics were subjected to thermal treatment at 120 °C for 48 h in oven to cross-link. The cross-linking between adjacent NMA moieties occurs by first stage removal of water forming bismethylene ether, followed by removal of formaldehyde forming a methylene bridge, as reported by Brown et al. [26]. Figure 1 shows the optical microscopic image of uncoated and coated fabric.

### 2.4. Characterization

^1^H NMR spectra were obtained by a Bruker Avance III, HD-600 MHz spectrometer (Bruker, Billerica, Massachusetts, USA) using d-dimethyl sulfoxide (DMSO-*d*_6_) as solvent in digital quad detection (DQD) acquisition mode. NMR was executed at 27 °C. A 5 mm probe was used. Delay time was 2 s. Chemical shifts (δ) were given in parts per million (ppm). FTIR spectroscopy was executed by FTIR spectrometer (JASCO FT/IR-4600, JASCO, Tokyo, Japan), to identify the bonds of functional groups, scanned over the range of 4000–500 cm^−1^ with spectral resolution of 4 cm^−1^ and 64 scans for each spectrum. We used powder sample ground in mortar-pestle without potassium bromide or any other alkali halide and dried for 1 h before use in oven at 60 °C. The thermal behavior of copolymers was investigated by a thermogravimetric analyzer (Perkin Elmer TGA-4000, Perkin Elmer, Waltham, MA, USA) from 30 °C to 700 °C at a heating rate of 10 °C/min under dry nitrogen flow rate of 20 mL/min. The LCST of the copolymers in the aqueous solution was monitored and recorded by the transmittance of a 600 nm light beam on a UV-Vis spectrophotometer (JASCO V-670, JASCO, Tokyo, Japan) with a temperature control device. The concentration of the copolymer in water was 1 mg/mL. The temperature was raised from 25 °C to 40 °C with a rate of 1 °C increment every 5 min. In the plot, the LCST corresponds to a 50% decrease in the initial transmittance. A swelling test was performed to confirm the cross-linking. The samples were dried at 100 °C for 2 h to remove moisture. The swelling ratio is the difference of the final and initial weight over the initial weight, and it is expressed in percentage. The surface wettabilities of the coated samples were characterized by a contact angle analyzer (Dataphysics OCA 15EC, Dataphysics, Filderstadt, Germany) using the sessile drop method at 20 °C (below the LCST) and 50 °C (above the LCST). A moisture absorption and release test was executed in a temperature-and-humidity controlled oven (TERCHY, T6800, Terchy, Taipei, Taiwan). Initially, we determined the dry weight of the samples. Further, the samples were treated with 65%, 95%, and again 65% relative humidity for 24 h each at 20 °C and 37 °C. After every 24 h, the weight of the samples was recorded. The weight change due to the humidity alteration from 65% to 95% over the dry weight determined the absorption rate. Similarly, the weight change from 95% to 65% over the dry weight determined the release rate. A mechanical property test was executed in a universal test machine (COMETECH QC-508M2, Cometech, Taichung, Taiwan) at room temperature according to ASTM D882. The dog-bone shaped specimens were prepared with a gauge length of 55 mm, width of 5.38 mm and thickness of 0.5 mm. Cross speed was 200 mm/min.

## 3. Results and Discussion

### 3.1. NMR Spectroscopy

**Figure 2:**
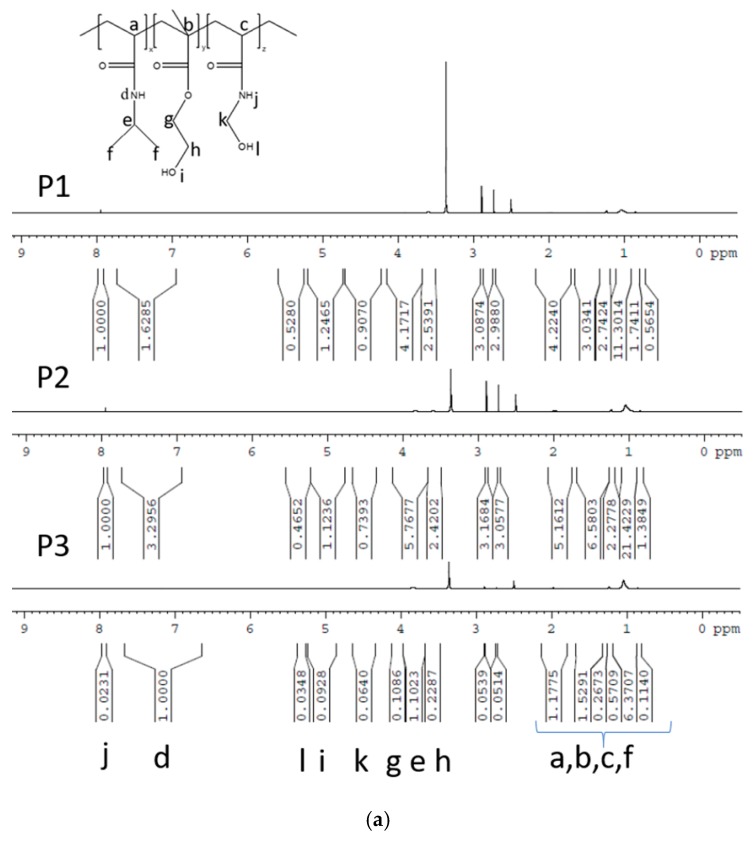

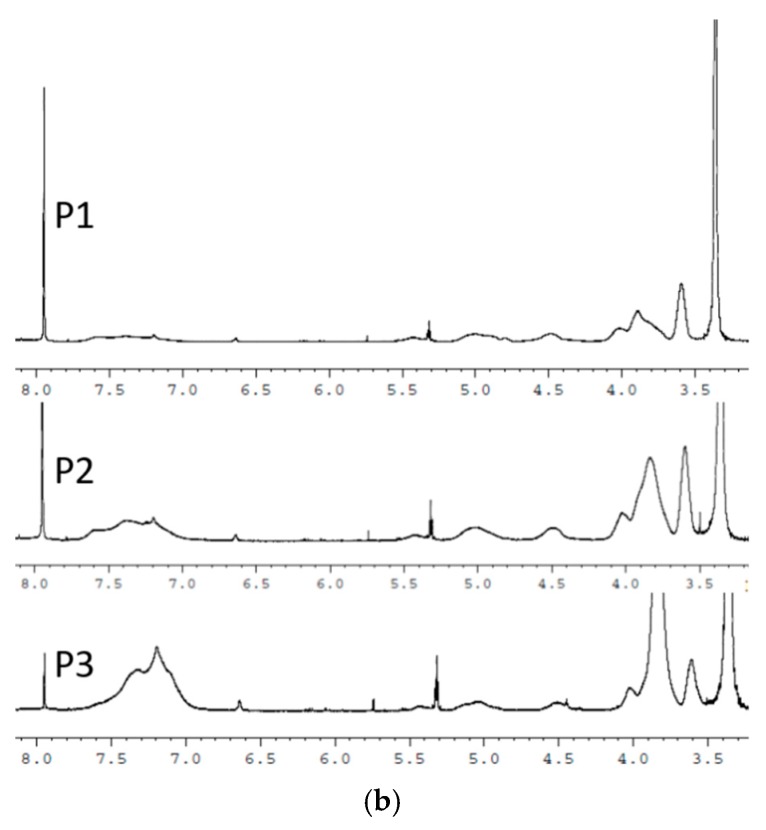
(**a**) NMR spectra with integral values and (**b**) major peaks of moieties in P1, P2, and P3 copolymers.

Figure 2a,b show the NMR spectra with integral value and proton peaks of the P1, P2, and P3 samples of poly(NIPAAm-*co*-HEMA-*co*-NMA), respectively. A chemical shift of 2.5 and 3.3 ppm are for the solvent DMSO-d6 and H_2_O, respectively. Overall major peaks from moieties are within 3.5 to 8.0 ppm. The shifts of NIPAAm, HEMA, and NMA are assigned as follows: At 7.1–7.6 ppm (d) and 3.8–4.0 ppm (e) are for secondary amine and methine neighbor of the methyl group in NIPAAm, respectively. Shifts at 4.8–5.1 ppm (i), 3.5–3.6 ppm (h), and 3.9–4.1 ppm (g) denote the terminal hydroxyl, the methylene neighbor of hydroxyl, and the methylene neighbor of the oxygen in HEMA, respectively. At 5.3–5.5 ppm (l), 7.9 ppm (j), and 4.4–4.7 ppm (k) correspond to the hydroxyl, secondary amide and alkyl chain of NMA, respectively. However, at 7.9 ppm, the hydrogen peak for secondary amine does not show in Figure 2b because a sharp DMF signal hides the peak at the same location. The peak shifts at 0.7–2.1 ppm (a, b, c, f) correspond to the terminal methyl of NIPAAm and the alkyl chains of the backbone. All the peaks of the three moieties reveal successful polymerization and the experimental molar ratios are close to the feed molar ratios.

### 3.2. FTIR Spectroscopy

**Figure 3:**
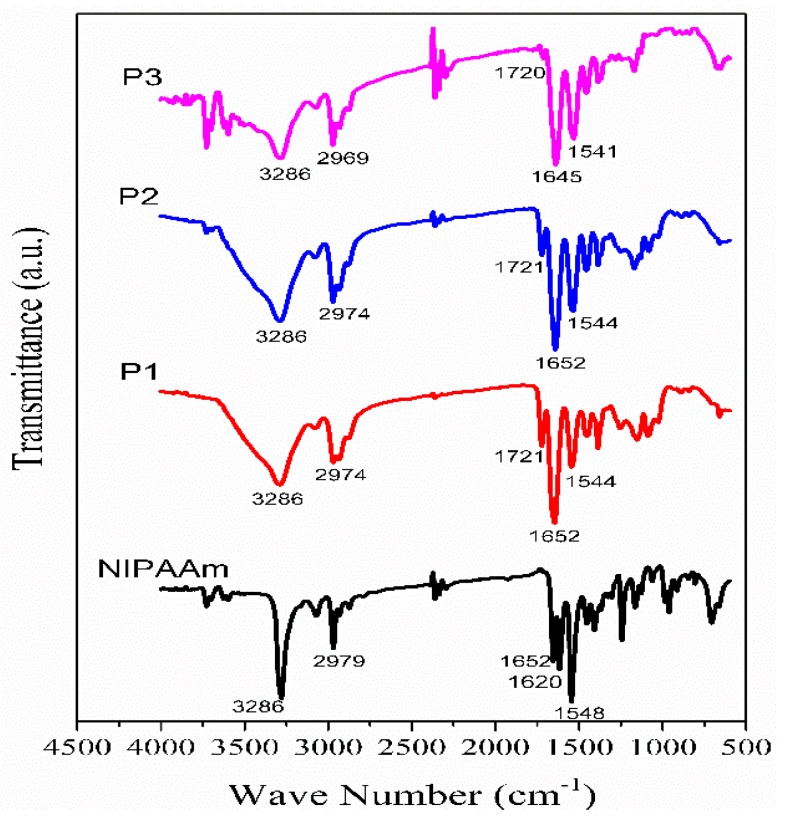
FTIR images of the NIPAAm monomer, and the P1, P2, and P3 copolymers.

Figure 3 shows the FTIR spectra of the NIPAAm monomer, and the P1, P2, and P3 copolymers. NIPAAm exhibits a characteristic peak at 3286 cm^−1^ and 2979 cm^−1^, responsible for N–H stretching and –CH_3_ asymmetric stretching, respectively. The absorption peak at 1548 cm^−^^1^ is attributed to the bending of the secondary amide, whereas the peaks at 1652 cm^−1^ and 1620 cm^−1^ are assigned to the stretchings of C=O and C=C. The FTIR spectra of the copolymers exhibit the disappearance of the peak at 1620 cm^−1^. This disappearance indicates the breaking of the C=C double bond, affirming successful polymerization. For the NIPAAm monomer, the absorption peak at 3286 cm^−1^ is sharp. However, after the polymerization, the peak is broadened because it overlaps with the peaks of the O–H stretching of HEMA and NMA at 3500 cm^−1^ and 3450 cm^−^^1^. Similarly, the peak of NIPAAm at 1652 cm^−1^ is broadened because of the C=O stretching of NMA. However, the characteristic peak of HEMA at 1721 cm^−1^ responsible for C=O stretching clearly appears, whereas it is not seen in the FTIR spectra of the NIPAAm monomer. The peak at 1544 cm^−1^ in the polymer spectrum denotes the bending of an N–H bond.

### 3.3. Thermal Behavior

**Figure 4:**
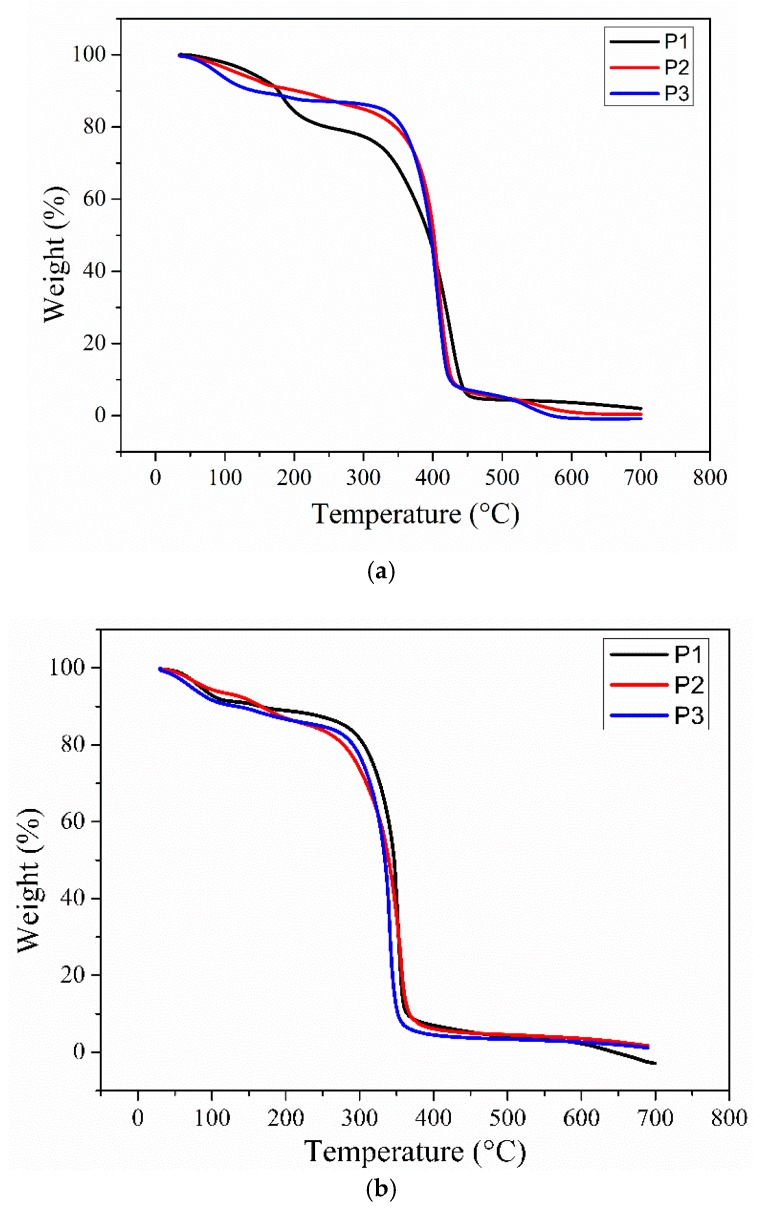
TGA curves of the P1, P2, and P3 copolymers (**a**) before and (**b**) after cross-linking.

Figure 4a shows the thermal behavior of the copolymers, in terms of the change of weight change percentage over temperature. Three stages of weight loss can be observed. From room temperature to approximately 150–180 °C, the gradual weight loss is mainly caused by evaporation of the retained water. From 320 °C to 430 °C, the rapid weight loss is caused by the degradation of side chains. From 430 °C onwards, the almost negligible weight loss indicates the degradation of the backbone chain.

The HEMA and NMA moieties in the side chains of the copolymers are highly endowed with a hydroxyl functional group, which is thermally labile [27]. Accordingly, the thermal stability of P1 is significantly lower than that of P2 and P3; and P1 is showing the lowest onset decomposition temperature at 321 °C with a higher molar ratio of HEMA and NMA than that of P2 at 336 °C and P3 at 346 °C. Moreover, the higher weight loss of P1 due to water evaporation from room temperature at 150–180 °C, compared to those of P2 and P3, agrees with the higher water absorption and retention property at high temperatures. Conversely, P3 (which has the lowest HEMA molar ratio) shows a minute weight loss initially due to water evaporation; but at a slightly higher temperature, the curve becomes almost horizontal until the onset of the decomposition temperature depicts lower water retention. However, all three copolymers exhibited major degradation of side chains between 320 °C to 430 °C. Among the side chains, hydroxyl functionalized HEMA and NMA started degradation approximately within 350 °C. NIPAAm moiety is comparatively more thermally stable as it started degradation later, around 400 °C. Backbones are more thermally stable as they degraded after 430 °C.

Figure 4b shows the thermal behavior of the copolymers after cross-linking. We can observe clearly that the onset decomposition temperature of P1 is higher than P2 and P3, which is completely opposite to the thermal behavior before cross-linking. This is due to the presence of a higher molar ratio of NMA in P1. Most of the terminal hydroxyl groups from NMA are involved in formation of a methylene bridge for cross-linking. Therefore, a number of such thermally labile, free hydroxyl groups are significantly reduced in P1 compared to P2 and P3. For P2 and P3, the thermal behavior is not significantly affected by cross-linking.

### 3.4. LCST

**Figure 5:**
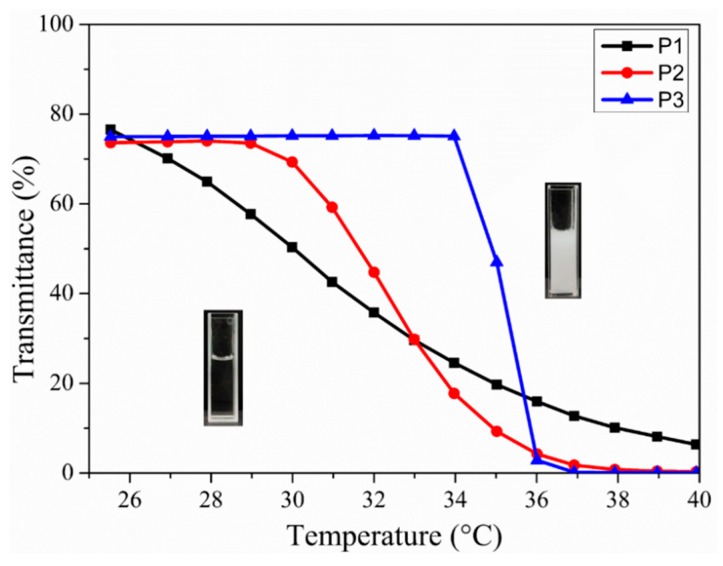
Variation in optical transmittance of P1, P2, and P3 copolymers.

Figure 5 shows the variation in optical transmittance over temperature. The evaluated LCSTs of P1, P2, and P3 are 31.0, 32.5 and 35.5 °C, respectively. The aqueous solution turned from transparent to white turbid as a result of a coil-to-globule conformational transition, in which the increasing temperature causes a hydrophilic to hydrophobic phase change, eventually forming precipitation.

Below the LCST, carbonyl groups of NIPAAm form intermolecular H-bonds with water molecules; thus, NIPAAm becomes hydrated, swollen, and eventually dissolved in water. Conversely, above the LCST, the H-bonds break and release water, facilitating polymer–polymer interactions by forming intramolecular H-bonds. Based on this mechanism, we designed the copolymer with HEMA and NMA to tune the LCST for thermoregulatory and moisture management applications. We noticed an increase in the LCST with a decrease in the HEMA molar fraction. P3 (which had the lowest molar fraction of HEMA) exhibited the highest LCST at 35.5 °C, which was close to the human body temperature. The higher tendency of the carbonyl and hydroxyl groups of HEMA to form H-bonds with the amide group of a neighboring NIPAAm triggers the release of water [28]. Zhang et al. reported a similar LCST behavior for an aqueous solution of a copolymer and Gan et al. for a microgel against volume phase transition temperature [29,30]. The P1 curve shows the gradual change, but the P3 curve is quite sharp because P3 contains less NMA. NMA with NIPAAm shows a hydrophilic nature that may retain some water molecules in P1 and release them gradually [31].

### 3.5. Swelling Test

For the swelling experiment, copolymers P1, P2, and P3 could swell in DI water for 24 h at 20 °C. The samples achieved different degrees of swelling. Table 1 elaborates on the degree of swelling for the three samples. P2 showed the highest degree of swelling. However, P3, even with the highest NIPAAm molar fraction, exhibited the lowest degree of swelling. The higher entanglement concentration of NIPAAm could have decreased the elasticity and hindered the water uptake. Gallagher et al. observed and explained such a decrease in swelling after increasing the PNIPAAm in a semi-IPN hydrogel [32]. The swelling test depicted that the cross-linking was successful in all the samples.

### 3.6. Surface Wettability

**Figure 6:**
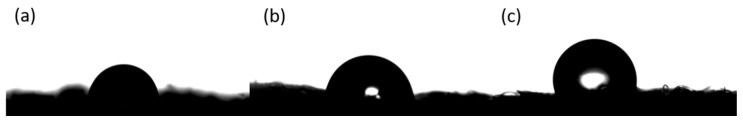
Water contact angle in air above the lower critical solution temperatures (LCSTs) of (**a**) P1, (**b**) P2, and (**c**) P3 samples.

Table 2 illustrates different contact angles in different conditions revealing surface wettability. The water contact angles of the samples were measured in air. When the temperature was lower than the LCST of the copolymer, all the samples exhibited superhydrophilicity with ~0° contact angle. This property helps to absorb moisture rapidly from the human body. Above the LCST, P1 and P2 showed an increase in contact angle at 76° and 78°, respectively. P3 exhibited the highest rise at 102°, as shown in Figure 6. The higher molar fraction of NIPAAm in P3 enhanced such hydrophobicity because of the formation of intramolecular H-bonds resulting in the repulsion of water. P1 and P2 could hardly absorb water even at 50 °C, as their contact angles were below 90°. Moreover, contact angle is closely related to surface tension which can be a cohesive force between molecules of liquid droplet or as adhesive force (interfacial tension) between liquid and solid molecules. Below LCST, all the three sample surfaces had high interfacial tension or surface energy which overcame the cohesive force of liquid, thus, showing high wettability. The surface energy measured at this condition was 73.43 mN/m for all three samples. However, above LCST, it decreases as the contact angle increases. Surface energy measured for P1, P2 and P3 samples were 36.79, 35.70 and 28.21 mN/m, respectively. This indicated that interfacial tension becomes lower above LCST and that spreading out of the liquid droplet was hindered.

This switchable wettability of our coated fabric enhanced the applicability compared to uncoated PET fabric. Chen et al. also achieved similar behavior with P(NIPAAm-co-NHMAAm) system for smart textile applications. However, change of contact angle was limited between 30 to 70° [15].

We also measured the oil contact angles in air for all the samples; they showed a high affinity towards oil below and above the LCST. All the samples can be considered as superoleophilic. Therefore, the wettability change in air can only be distinguished by considering the water contact angle. Wang et al. discovered a similar oleophilic behavior for an NIPAAm-based hydrophilic membrane [33]. However, their PNIPAAm-RC nanofibers show a superhydrophobic nature above LCST which is not suitable for our moisture management application due to lesser tendency of water absorption at this condition.

The water contact angle under oil stayed almost unchanged below and above the LCST. The coated polymer tried to absorb oil into the pores of the fabric and increased the surface roughness heterogeneously because of its superoleophilicity. When water was dropped onto the surface, the intermediate oil layer delayed the spreading more than air, due to its heterogeneous surface. This delay increased the value of the contact angle for all the samples. However, the contact angle of water decreased with time. Oil molecules penetrated the fabric pores, altering the heterogeneous surface to a homogeneous surface because of the oil affinity. The droplet spreading was much slower than that in air. Ranganath et al. reported an initial high contact angle that decreased with time under oil media, as well [34]. They studied a wide range of wettability in air, water and oil media with a PNIPAAm-PVDF blend/PVDF system. We investigated wettability in such media with our coated fabric, additionally considering the temperature above LCST.

The underwater oil contact angle suggests that the oleophobic nature of the surface is unaffected by the LCST. As the porous fabric surface was coated with the polymer solution, there was some surface roughness. According to the wetting theory, a Cassie state was introduced on the heterogeneous surface. The surface canyons entrapped water below the LCST because of hydrophilicity. When oil approached the surface, entrapped water molecules repelled it, making the surface oleophobic. When the surface becomes hydrophobic above the LCST, the molecules of water come out of the pores immediately above the coating layer. However, the temperature is not high enough for the water to evaporate; hence, the oil droplet is repelled by the relatively hot water layer again. This solid–liquid (water)–liquid (oil) interface behaves similarly to the surface of red roses with micropapillae and nanogrooves, and can be applied for self-cleaning [35]. Further, the surface tension of oil is much lower than that of water. This also helps to increase the oleophobicity.

### 3.7. Moisture Management

The moisture absorption and release of all the samples were enhanced at 20 °C and 37 °C compared to those of the bare fabric. However, both absorption and release decreased with increasing temperature, as shown in Table 3. Below the LCST, all the samples showed high absorption rates. This phenomenon can be explained as the moisture uptake is governed by diffusion and absorption [36,37]. The moisture absorption depends on the formation of H-bonds between water molecules and NIPAAm. However, the moisture release is governed by concentration-dependent diffusion. When humidity decreased from 95% to 65%, water vapor was released to the surrounding air to maintain a constant vapor concentration. In textile applications, this release mechanism corresponds to the release of insensible perspiration. For the saturated moisture in the fabric, the predominant mechanism is capillary action correlating to the release of sensible perspiration. The equilibrium of moisture concentration between the microclimate and the surrounding air is manifested in both cases. Above the LCST, the absorption was governed only by concentration-dependent moisture diffusion, lower than that below the LCST. Additionally, the release rate was quite high because NIPAAm broke some H-bonds generating free water vapor molecules. The higher temperature also provided more energy for the diffusion of water vapor to the surrounding air. This response is highly beneficial for reducing the humidity of a microclimate rapidly, giving comfort to the human body during sports or physical exhaustion. Regarding the bare PET fabric, the absorption and release rates increased with increasing temperature because higher temperature accelerated the diffusion of water molecules within the fabric. Table 3 illustrates the amounts of moisture absorption and release.

P2 exhibited the best absorption and release at both temperatures. Although all samples exhibited superhydrophilicity below their LCSTs, P2 showed the highest amount of water uptake in the swelling test. P3 exhibited better absorption and release than P1, owing to the formation of more H-bonds with water molecules, along with concentration-dependent diffusion. P2 performed best above the LCST because it was still hydrophilic with a 78° water contact angle and absorbed the highest moisture content by diffusion. However, P3 showed lower absorption and release than P1, as the mechanism of absorption by the formation of H-bonds could not be predominant here. Rather, the higher hydrophobicity of P3 made it the least moisture-absorbing/releasing material at 37 °C.

### 3.8. Mechanical Property

**Figure 7:**
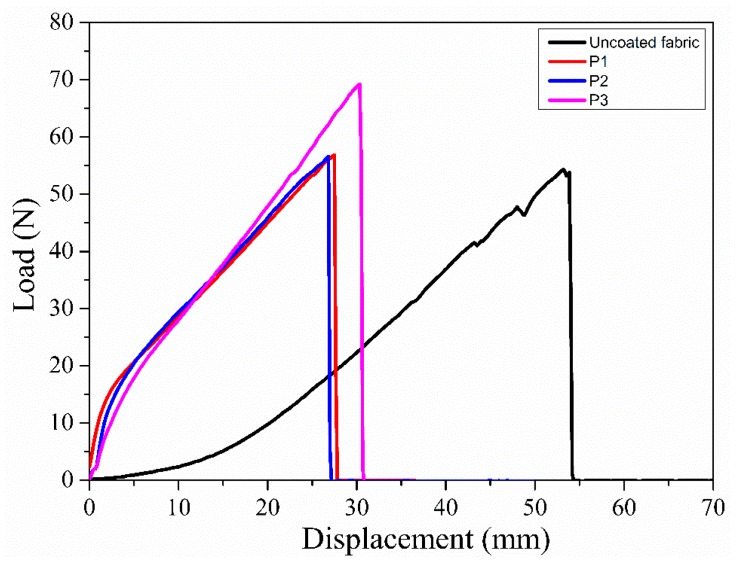
Load-displacement curve of uncoated fabric and P1, P2, P3 coated fabric.

The load-displacement curves of uncoated fabric and P1, P2, P3 coated fabric are shown in Figure 7. The maximum load for uncoated fabric was 54.55 N and that for P1, P2 and P3 coated were 56.89, 56.89 and 69.46 N, respectively. Coated samples showed a comparatively higher maximum load than uncoated fabric. Failure displacement of uncoated fabric and P1, P2, P3 coated fabric were 53.23, 27.46, 26.81 and 30.38 mm, respectively. It can be seen that failure displacement was significantly reduced in coated samples. Coated samples became more stiff than the uncoated samples. This manifests the loss of ductility and embrittlement due to the polymer coating layer. Among coating samples, P1 and P2 did not show any significant difference. P3 showed little higher maximum load and failure displacement. Overall maximum load was not different in samples because the main responsible component for carrying load was fiber which remained unchanged after coating.

## 4. Conclusions

A smart thermoregulatory material with moisture management capabilities was successfully produced as poly(NIPAAm-*co*-HEMA-*co*-NMA) coated PET fabric. The LCST of the copolymer was tailored by tuning the molar ratio of the moieties. The surface of the material exhibited switchable wettability under different conditions. Further, the material showed a change from a state of high moisture absorption/release below the LCST to a state of low moisture absorption/release above the LCST. This variation in moisture management can balance the humidity of a microclimate with the surrounding air and provide comfort to the human body. The P2 coated PET fabric exhibited the best moisture absorption/release results. This material has the potential for smart thermoregulatory moisture-control applications.

## Figures and Tables

**Table 1:** Compositions, thermal, and swelling properties of copolymers. LCST: lower critical solution temperature.

Sample	Feed Molar Ratio (%) NIPAAm:HEMA:NMA	Experimental Molar Ratio (%) NIPAAm:HEMA:NMA	*T*_d_ (°C)	LCST (°C)	Degree of Swelling (%)
P1	50:37.5:12.5	48.9:37.5:13.6	321	31.0	1153
P2	70:22.5:7.5	68.8:23.5:7.7	329	32.5	2200
P3	90:7.5:2.5	88.9:8.3:2.8	341	35.5	687

**Table 2:** Contact angles in different conditions.

**Sample**	**WCA in Air**		**OCA in Air**	
	**Below LCST**	**Above LCST**	**Below LCST**	**Above LCST**
P1	0°	76°	0°	0°
P2	0°	78°	0°	0°
P3	0°	102°	0°	0°
Sample	WCA under oil		OCA under water	
	Below LCST	Above LCST	Below LCST	Above LCST
P1	81°	89°	92°	105°
P2	91°	106°	119°	100°
P3	116°	115°	134°	114°

WCA: Water Contact Angle; OCA: Oil Contact Angle.

**Table 3:** Moisture absorption and release at different temperatures.

Sample	20 °C		37 °C	
	Absorption (%)	Release (%)	Absorption (%)	Release (%)
P1	5.04	3.40	2.41	2.09
P2	7.48	5.68	3.27	3.14
P3	6.30	3.78	2.17	1.91
PET	0.25	0.25	0.26	0.26
